# Recent Advances in Management of Pediatric Food Allergy

**DOI:** 10.3390/children2040439

**Published:** 2015-12-15

**Authors:** Katherine Anagnostou, Kate Swan, Adam T. Fox

**Affiliations:** Department of Paediatric Allergy, Guy’s and St Thomas’ Hospitals NHS Foundation Trust, Westminster Bridge Road, London SE1 7EH, UK; E-Mails: Aikaterini.Anagnostou@gstt.nhs.uk (K.A.); Kate.Swan@gstt.nhs.uk (K.S.)

**Keywords:** food, allergy, children, immunotherapy, probiotics

## Abstract

Many children now suffer with a food allergy, immunoglobulin E (IgE) and/or non-IgE mediated. Food allergies have a significant impact on the child’s quality of life, as well as that of their family, due to the resultant dietary restrictions and the constant threat of a potentially life-threatening reaction. At present, there is no cure for food allergies, but there are exciting advances occurring in the management of IgE mediated allergies, including a more active approach to management with anticipatory screening testing, early introduction of common food allergens, active tolerance induction, use of biologics and active risk management. These areas will be discussed in this review.

## 1. Introduction

Food allergy prevalence has been increasing over the last few decades; currently, it affects 6%–8% of children. This increase in prevalence has also been mirrored in other atopic disorders in the Western world [1,2]. The highest prevalence is seen in the younger infants and toddlers with 2.5% of infants suffering from milk allergy and up to 10% of one-year-olds having to live with a range of food allergies, such as cow’s milk, egg, nut, soya, wheat, fish and shellfish [3,4,5].

There is known to be a significant impact on quality of life from all atopic diseases and food allergy is no exception. Indeed, food allergy sufferers are reported to have a lower quality of life than insulin-dependent diabetics [6]. This is, in part, due to the fear and anxiety that results from the threat of accidental ingestion and resultant potential life-threatening reactions, but also due to the social restrictions that occur secondary to the dietary restrictions and may cause issues at school or college [6].

Although there is abundant data demonstrating that many food allergies, in particular, cow’s milk, egg, soya and wheat are outgrown, there is currently no cure for food allergy [7,8]. Fish, shellfish, peanut and tree-nut allergies are rarely outgrown and tend to be lifelong [9]. The traditional approach to managing food allergies has, therefore, been strict elimination diet and treatment of adverse reactions due to accidental ingestion. This is guided by written allergy action plans given to the children and their families along with the required emergency medications, mainly adrenaline, antihistamine and short acting β-adrenergic agonists in inhaler form [10].

Recent advances in the management of food allergy have resulted in the adoption of a more active approach. Previously, it was not always deemed important to actively test for related allergens, once a specific food allergy was identified, but this screening/preventative testing has become more common practice. A particularly exciting and recent advance has been the increasingly evidence-based practice of early introduction of potentially allergenic, but tolerated, foods into the diet of infants, in order to prevent the development of allergy. Active induction of tolerance to known allergens has many facets and includes the role of adjuncts, such as probiotics and biologicals. Finally, risk management has become more active in nature. These recent developments rely on an emerging evidence base and although they may increase the complexity of managing children with food allergy, they have exciting implications for better quality of life and for allergy prevention.

## 2. Screening for Further Food Allergies

The presentation of a child with a food allergy indicates that further testing and assessment is required, due to the known risk of that child having other food allergies, both related and unrelated to the presenting allergen [11]. One approach would be to leave the child to discover further allergies, when he or she encounters an allergen and reacts, but this could have potentially severe consequences. Another approach would be a blanket ban on consuming foods considered to be “high risk” such as nuts, due to the fear of a possible reaction. This further fuels anxiety and adds to the burden of food avoidance, often unnecessarily, such as the avoidance of shellfish in children with fish allergy.

The recent National Institute for Health and Care Excellence (NICE) guidelines for food allergy recommend screening for known co-allergens, as well as testing to the suspected index reaction food allergen [12,13]. This anticipatory testing involves skin prick or specific immunoglobulin E (IgE) testing and if there is diagnostic doubt, an oral provocation challenge [10]. Co-allergens are allergens known to occur with increased frequency in those with certain other allergens. Peanut allergy is seen in 20%–30% of children with egg allergy and sesame allergy has an estimated prevalence of 25% in those with peanut allergy [14,15].

There is also a relationship between eczema and food allergy with extensive evidence, from over the last 15 years, that eczema in infancy is an important risk factor for the development of IgE-mediated food allergy. Increasing severity of eczema and earlier age at onset are both risk factors for developing food allergy [16,17,18,19,20]. More recently, a study by Martin *et al.* [21] used oral food challenges in children aged 11 to 15 months with eczema and positive skin prick tests to egg, peanut and sesame to demonstrate that infants with eczema were 11 times more likely to develop peanut allergy and 5.8 times more likely to develop egg allergy by 12 months compared to those with no eczema. Infants aged 0 to 3 months, with eczema requiring prescription only topical corticosteroids, had a greater than 50% chance of developing a combination of peanut, egg and sesame seed allergies. Screening for food allergens should therefore be extended to these patients, ideally before they are even exposed to allergenic foods, at the time of weaning. The testing can be focused onto those foods that are known to be commonly allergenic and relevant for the child’s ethnic background and household diet, such as milk and egg, which are common around the world or chickpea and lentil, for families with a South Asian diet, in whom these allergies are more common [22]. A thorough evaluation may enable early introduction of allergenic foods, which has the potential to reduce the risk of allergy developing, as will be discussed later.

Central to this anticipatory approach is the ability to differentiate between allergy and sensitization, especially in children with no clinical history of consuming the suspect allergen. Ongoing improvements in allergy diagnostics may aid with this process. Component-resolved diagnostics may help to better differentiate between sensitization and clinical reactivity and reduce the need for food challenges. Proof of principle has been demonstrated for milk, egg, peanut and various other allergens [23,24,25,26,27,28,29]. Components in peanut allergy are particularly interesting: Ara h 2 has been shown to have increased diagnostic value over peanut specific IgE, but more studies are needed due to differing correlations between values and outcomes seen in different populations and geographical regions. One recent study, demonstrating geographical variance in immunological reactivity to peanut components, showed that American patients had higher levels of IgE antibodies to Ara h 1, Ara h 2 and Ara h 3, whereas patients from Spain had higher rates of sensitization to Ara h 9 and Swedish patients had higher levels of IgE antibodies to Ara h 8 (the Bet v 1 homologue) [30].

Despite these advances in diagnostics, allergy screening will inevitably lead to an increased burden on food challenge services, when there is diagnostic uncertainty with no history of consumption or exposure to guide the clinician. Food allergy often resolves over time: milk allergy has been shown to resolve in 79% by the age of 16 years [7] and 68%–82% of children outgrow their egg allergy by 16 years [8]. There is, therefore, a role for monitoring food-specific IgE antibodies (decreasing levels are associated with an increased chance of allergy resolution) to determine when a food challenge should be repeated [31]. Repeating food challenges at regular intervals to check for resolution, can have important benefits for the allergic child and the family [32]. Re-introduction of the food does not only have nutritional benefits, but also the potential to significantly improve quality of life [33,34]. However, it is important to note that food challenges are not without risk and have a significant resource implication [35,36].

In summary, anticipatory testing for related co-allergens, in carefully selected patients would allow more precise and prompt diagnosis and reduce the risk of unnecessary avoidance of safe foods or reactions to unknown allergens. The health economics of the resultant increased testing, follow-up appointments and food challenges needs to be further evaluated.

## 3. Early Introduction of Allergenic Foods

The LEAP (Learning Early About Peanut allergy) study has thrown this area into the spotlight very recently, providing strong (Level 1) evidence that, for peanut at least, early introduction in “at-risk” infants is safe and can markedly reduce the likelihood of developing peanut allergy. Almost two decades prior to this, in 1998, the UK Department of Health and the American Academy of Pediatrics, concerned at the rise in incidence of peanut allergy, issued guidance on consumption of peanuts during pregnancy and lactation. These stated that, “pregnant women who are atopic or have an atopic partner may wish to avoid eating peanuts during pregnancy and lactation. Infants with a family history of atopy, should be exclusively breastfed for 4–6 months and should avoid peanuts until 3 years” [37]. This advice was withdrawn in 2009 due to lack of evidence of any benefit from maternal avoidance in preventing the development of food allergies [38]. Studies investigating whether increased consumption of nuts during pregnancy was protective were also inconclusive with contradictory findings of reduced risk or no significant association [39].

The guidance regarding delayed introduction of solid foods until 6 months and allergenic foods until after 3 years in at risk infants with family history of atopy has also been challenged. There has been a gathering of momentum over recent years towards acceptance that early introduction of allergenic food may be important for inducing tolerance as evidenced by several studies [40].

To date, milk, egg and peanut have been the most thoroughly investigated with regards to early allergen exposure, but this year will bring us the first results from the EAT (Enquiring About Tolerance) study (trial registration number: ISRCTN14254740 DOI 10.1186/ISRCTN14254740), which is testing the hypothesis that the introduction of six allergenic foods (cow’s milk, peanut, wheat, egg, sesame and fish) into the diet of infants from 3 months of age, alongside continued breastfeeding, results in a reduced prevalence of food allergies by three years of age. It is a large randomized controlled study—involving 1302 participants—with the intervention group introducing the six allergenic foods and the controls following current UK government advice of aiming to exclusively breastfeed until approximately 6 months, with no introduction of the above allergenic foods before 6 months.

Interest in the early introduction of peanut was prompted by the findings from the study by Du Toit *et al*. [15], who found a 10-fold higher prevalence of peanut allergy among Jewish children in the UK compared with those in Israel (1.85% *versus* 0.17%). The effect of genetics was unlikely to account for the difference in prevalence because the participating children shared a similar genetic background. However, it was noted that Israeli infants start consuming peanut-containing foods during early weaning, whereas UK infants mostly avoid peanuts for the first three years of life. The investigators concluded that early consumption of peanuts in infancy, as well as consumption of frequent and high doses of peanut protein is associated with a low prevalence of peanut allergy, possibly due to induction of oral tolerance. This study was observational and the investigators looked to design an intervention study that could answer this hypothesis more rigorously—the LEAP study.

The LEAP study was a prospective, randomized, controlled trial involving the randomization of 640 patients aged between 4 and 11 months with severe eczema and/or egg allergy to either consumption or avoidance of peanut. There was an 86% reduction (13.7% in avoidance group *versus* 1.9% in consumption group) in peanut allergy prevalence at 60 months in the participants with negative skin prick tests at baseline in the intention to treat analysis and a 70% reduction (35.5% to 10.6%) in those who had a skin prick test at study entry of between 1 to 4 mm to peanut. Combining both cohorts, there was an 80% relative risk reduction, a 14% absolute risk reduction and the number needed to treat to prevent peanut allergy was 7.1 [41]. These results indicate a real possibility of preventing peanut allergy (both primary and secondary prevention), therefore, The World Allergy Organisation (WAO) has published interim guidance based on the evidence from this study in which it is stated that “healthcare providers should recommend introducing peanut containing products into the diet of ‘high risk’ infants early on in life (between 4–11 months of age)” and “infants with early onset atopic disease, such as severe eczema, or egg allergy in the first four to six months of life may benefit from evaluation by an allergist”. It was also suggested that skin prick testing should be considered by the allergist and in-office observed peanut ingestion may be required for those patients with evidence of peanut sensitisation on skin prick test. (www.worldallergy.org/consensus-communication-early-peanut-introduction) On the LEAP study, the infants consumed 2 g of peanut protein three times a week and it is not known whether different doses would have the same outcome. It is also not known whether tolerance or temporary desensitization has been induced, but the LEAP-ON study, which is currently underway, aims to address this question. In the LEAP-ON study, the patients are asked to stop consumption of peanut for 1 year, after previous continuous consumption of 5 years, followed by a repeat challenge. This data will be available soon.

Early exposure to cow’s milk has been analyzed in a large cohort of over 13,000 infants, as part of a prospective study investigating risk factors for cow’s milk allergy. Katz *et al.* [42] found that infants with exposure to cow’s milk protein in the first two weeks of life had a significantly lower incidence of cow’s milk allergy compared with those infants in whom cow’s milk was only introduced after the age of 4-6 months. The investigators concluded that early exposure to cow’s milk protein may be protective against the development of IgE mediated cow’s milk allergy.

Early introduction of egg was studied in a cross-sectional study by Koplin *et al.* [43]. The study indicated that early introduction of egg might have a protective effect against egg allergy. The study included 2589 infants and examined the relationship between timing of infant feeding and subsequent risk of food allergy. Infants introduced to cooked egg at 4-6 months had a lower risk of egg allergy than those introduced to cooked egg after that time. In Finland, a birth cohort of 3781 children was studied by Nwaru *et al.* Egg introduction before the age of 11 months was inversely associated with the development of asthma, allergic rhinitis and atopic sensitization. In contrast, introduction of a limited number of foods at the age of 3 months was associated with an increase in atopic sensitization later in life [44,45].

Overall, the evidence is growing to support the view that introduction of allergenic foods should not be delayed and where there is an increased risk of co-allergy, e.g., for peanut amongst egg allergic children, or increased risk of early development of food allergy, e.g., in infants with eczema, the use of anticipatory testing can help support the early introduction of allergenic food into the diet. This helps broaden the dietary repertoire of the infant and reduces the number of foods being avoided. The above approach is particularly useful as increasing number of food avoidances, has been shown to increase the risk of nutritional compromise and abnormal feeding patterns [46,47].

## 4. Active Tolerance Induction

After the diagnosis of a food allergy, the traditional approach has been strict avoidance, however, recent evidence has started to emerge, relating to new strategies to help tolerance develop more quickly [10,48]. Recent studies investigating the natural history of cow’s milk and egg allergy have shown that resolution rates are not as high as previously considered, with the majority of milk and egg allergic children only developing tolerance in late childhood [7,8]. Persistence of both allergies into late childhood years often affects nutritional status and quality of life. It would, therefore, be beneficial to find ways that would accelerate tolerance development. Three areas have been of particular interest—the use of probiotics in infants with milk allergy, the role of baked egg and milk introduction in children with milk or egg allergies and the role of desensitization to food.

The potential role for probiotics to modulate the allergic response in food allergy has been increasingly investigated over the last decade, in particular in milk allergy [49]. There is recent evidence that maternal probiotic supplementation during pregnancy and breastfeeding has been shown to reduce the risk of eczema in high-risk infants [50]. A study by Canani *et al.* took a group of 80 infants (age 1–12 months), who were randomly assigned to receive either an extensively hydrolyzed casein formula (EHCF) or EHCF with *Lactobacillus* GG (LGG) to investigate the role of LGG on acquisition of tolerance in infants with cow’s milk allergy. Infants receiving EHCF with LGG had a higher probability of acquiring tolerance to cow’s milk than those receiving ECHF alone, when challenged to milk at 6 months and 12 months. In conclusion, supplementation of ECHF with LGG was shown to accelerate the development of tolerance in infants with cow’s milk allergy [51].

Further evidence of an effect has come from a multicenter study observing the development of tolerance in 260 milk allergic children who had been started on a number of different hypoallergenic formulas [52]. The rate of acquiring oral tolerance after 12 months was significantly higher in the groups receiving EHCF (43.6%) or EHCF + LGG (78.9%) compared with the other groups: rice hydrosylate (32.6%), soy formula (23.6%), and amino acid formula (18.2%). The authors concluded that EHCF appeared to accelerate tolerance acquisition compared with other formula choices and that this effect is augmented by LGG [52]. However, the study was neither randomized nor interventional and hence open to selection bias, with more severe cases of milk allergy, most likely to run a prolonged course, more likely to have been prescribed amino acid formula. Interventional studies, which are underway, are required to clarify this effect of probiotics on tolerance induction.

This positive effect of probiotics has more recently been demonstrated in children with peanut allergy who are undergoing oral immunotherapy. A double-blind, placebo-controlled randomized trial involved 62 children who were randomized to either *Lactobacillus rhamnosus* (probiotic) and peanut oral immunotherapy (PPOIT) or placebo. PPOIT induced sustained unresponsiveness two to five weeks after stopping treatment in 82.1% compared to 3.6% receiving placebo [53]. This study does not compare peanut oral immunotherapy alone with PPOIT, therefore, it is not able to determine how much of the result was due to the addition of the probiotic, but it is an interesting approach requiring further research.

The incorporation of baked milk or egg has been recognized as influencing the development of tolerance to milk and egg. Despite a diagnosis of milk or egg allergy, many children can tolerate the allergen when extensively heated, such as baked in cakes, waffles or muffins. Studies revealed that 70%–75% of milk and egg allergic children can tolerate the allergen when extensively heated [54,55]. This can potentially help develop tolerance more quickly, as well as making dietary restriction much easier.

In one group of children who incorporated baked milk products into their diet, 59% of subjects developed tolerance to milk in the baked milk-consuming group compared with only 22% in the comparison group, who followed strict milk avoidance. In this study by Kim *et al.* [56], subjects who incorporated baked milk into their diet were 16 times more likely to become tolerant to all forms of milk than those in the comparison group. The addition of baked milk into the diet of these milk allergic children appeared to accelerate resolution of their cow’s milk allergies.

Extensively heated egg introduction shows a similar pattern. Another study evaluated the role of baked egg in the development of tolerance to regular egg in 70 subjects. Of those regularly consuming baked egg, 53% were tolerant of regular egg by the end of the 37-month study period, compared with only 28% (of the 47 subjects) in the comparison group (strict avoidance of all forms of egg). Subjects who were consuming baked egg products were 14 times more likely to develop tolerance to regular egg compared with the comparison group and on a faster timescale. The median time to regular egg tolerance was 50 months in the baked egg-consuming group *versus* 78.7 months in the comparison group [57]. The Healthnuts Study was a population based cohort study of over 5000 infants who had skin prick tests and food challenges to four allergens including egg. One hundred and forty of those infants with challenge proven egg allergy were followed up and the egg allergy resolved by two years in 56% of those who tolerated baked egg at one year, compared to 13% of those who continued to be baked egg allergic at one year [58]. There was also an association between the frequency of baked egg ingestion and the resolution of egg allergy at 2 years. Children eating baked egg at least five times a month were more likely to tolerate egg at 2 years, compared to those eating it four times a month or less (adjusted odds ratio 3.52) [58]. Unfortunately, it can be difficult to identify those children who can and cannot tolerate the allergen in the extensively heated form. Allergy testing, even with the use of component testing, is relatively unhelpful [55]. Reactions to baked egg or milk may be severe, hence it remains necessary to use oral food challenges under medical supervision to define the allergic status of each child before regular introduction into their diet [43,59].

Food oral immunotherapy (OIT) is another area of intense research interest and has shown promise as a form of active treatment for food allergies. The administration of small, but increasing doses of an allergenic food to children that are allergic to the food, has been shown to increase their clinical tolerance and enable them to eat varying amounts without reactions. Two different terms are often used in immunotherapy studies: desensitization and long-term tolerance. Desensitization refers to a raise in the threshold of reactivity to a specific food and requires regular consumption of the allergen, in order to be maintained. Long-term tolerance on the other hand, refers to ad lib consumption of the previously allergenic food, without need for regular treatment. Food oral immunotherapy studies have been conducted for various allergens, but mostly concentrated on milk, egg and peanut.

Skripak *et al.* [60] conducted a double-blind placebo-controlled study of OIT for cow’s milk allergy in twenty subjects 6–19 years of age. After OIT, the median cumulative dose inducing a reaction in the active treatment group was 5140 mg compared with 40 mg pre-OIT. There was no change in the median threshold in the placebo group. Although reactions were common during the study, nearly 90% were transient and did not require treatment.

A recent double-blind placebo-controlled, randomized egg oral immunotherapy study of 55 children, 5–18 years old, with egg allergy, resulted in a 55% rate of desensitization in the active group after 10 months of therapy. No subjects in the placebo group were desensitized. Once maintenance was discontinued for 6-8 weeks however, only 28% of children were desensitized at 24 months [61].

A two-step, phase 2, randomized-controlled crossover trial of peanut OIT in 99 children aged 7–16 years, investigated the role of peanut oral immunotherapy in peanut allergic children inclusive of all severities of peanut allergy. Among OIT participants, 84% were desensitized to 800 mg, the equivalent of five peanuts. In the active group, 24 of 39 (62%) OIT participants were desensitized to 1.4 g of peanut protein, compared with 0 of 46 participants in the control group. When compared with control participants, those successfully completing the study’s OIT protocol, had a significant 25-fold increase of their threshold level of reactivity to peanut over baseline. A significant improvement in the quality of life was reported for both participants and their caregivers. Despite 20% of all OIT participants reporting adverse events, reactions were mild and mostly treated with oral antihistamines and β2-adrenergic agonists [62].

Future studies are needed to further improve safety and efficacy of OIT. Families contemplating such therapy will need to balance the benefit of increased clinical tolerance with the risk of allergic reactions, compared to a strategy of complete avoidance. It is also important to think about the effects of co-factors, of which many cannot be controlled, on the acquired desensitization. Known co-factors include infection, exercise, anxiety and menstruation.

Overall, we can conclude that there is evidence to suggest that probiotics, as well as introduction of baked milk and baked egg into the diet of children allergic to cow’s milk and egg respectively, may have a role in active induction of tolerance. Furthermore, for those who do not naturally outgrow their allergies in early childhood, OIT is being seen as an increasingly realistic option, albeit still not in widespread clinical practice. Over time, management of food allergy has thus become a balancing act between avoiding allergens and promoting acquisition of tolerance [63].

## 5. Recent Advances in Advisory Labeling (Active Risk Management)

Current legislation dictates that, in many countries around the world, specific food allergens must be disclosed when they are ingredients in pre-packed foods, although it does not cover cross-contamination of food products. This legal requirement currently covers 14 food allergens. In addition, manufacturers can voluntarily choose to add advisory warning labels (AWLs) to warn about the potential of cross-contamination or “may contain” statements. [64,65].

The widespread use of AWLs, and the variability in wording employed (such as “may contain….”, “may contain traces of…” or “made in a factory….”), causes considerable confusion and anxiety to people with allergies and their carers [65]. A good proportion of foods with AWLs do not contain sufficient amounts of allergen to trigger a reaction in an allergic individual but the risk is greater in certain food products, such as confectionery items, [64] which can add to the consumer confusion. Worryingly, foods with no AWLs may, in fact, contain high enough levels of allergen to reach the threshold level for triggering allergic reactions in certain individuals [66,67,68], whose threshold may vary according to co-factors discussed earlier in relation to OIT.

Consumers who avoid products with AWLs spend 39% more time identifying appropriate foods and spend an additional 11%, on average, compared to their non-allergic counterparts [65]. A large proportion of allergic patients disregard AWLs, without seemingly experiencing adverse effects [69], despite many patient-support groups recommending complete avoidance in an understandable attempt to minimize the risk of reactions. Only approximately 50% of parents of nut allergic children avoid products with labels including “cannot guarantee is nut free”, “may contain traces of nuts” and “this product does not contain any nuts but is made in a factory that uses nuts”. Previous allergic reaction to nut products had no bearing on outcome [69]. Even among British health care professionals there is a marked variation in the advice provided to patients with allergies: a recent survey revealed that only 38% recommend complete avoidance of foods with AWL to nuts (but no nut listed in the ingredients), whilst 22% advised no avoidance was necessary. The majority took an individual approach and recommended avoidance only in certain circumstances, such as if they were unwell or didn’t have their emergency medication. A history of asthma or anaphylaxis increased the likelihood that complete avoidance was recommended [70]. This individual approach remains best practice whilst more data is required to better understand what, if any, additional risk results from less stringent avoidance.

## 6. Conclusions

There have been many recent advances in the field of pediatric food allergy that have enabled progress past the traditional management of food allergy, that consists of allergen avoidance and emergency treatment, while patients hope to outgrow their allergies. Now patients can look forward to greater knowledge of exactly what should be avoided (beyond the index reaction allergen) and by early introduction of high risk foods determined to be tolerated at the initial consultation, they may even be able to prevent the development of further allergies. For those with established allergies, there is encouraging progress with desensitization, as an exciting area of future development, to reduce risk of accidental reactions or even lead to long-term tolerance. All of these areas, along with advances in labeling and knowledge of non-IgE allergies will hopefully also result in improved quality of life for these patients. The health economic impact of these new approaches remains largely undefined.
